# THE ROLE OF APOEE4 IN THE ASSOCIATION BETWEEN PRODUCTIVE AND LEISURE ACTIVITIES AND COGNITIVE HEALTH

**DOI:** 10.1093/geroni/igae098.3753

**Published:** 2024-12-31

**Authors:** Yeonjung Lee, Ernest Gonzales, Yanyan Wu, Kathryn Braun, Peter Martin, Bradley Willcox, Ross Andel

**Affiliations:** University of Hawaii at Mānoa, UH Mānoa, Hawaii, United States; New York University, New York University, New York, United States; University of Hawaiʻi at Mānoa, Honolulu, Hawaii, United States; University of Hawaii, Honolulu, Hawaii, United States; Iowa State University, Ames, Iowa, United States; Kuakini Medical Center, Honolulu/Kuakini Medical Center, Hawaii, United States; Arizona State University, Phoenix, Arizona, United States

## Abstract

Cognitive health is influenced by genetic and environmental factors. This study fills a gap of knowledge by examining the role of the apolipoprotein E gene, APOEε4, within the context of how employment, civic engagement, and leisure activities relate to cognitive health. Using pooled data from the Health and Retirement Study (HRS) Psychosocial and Lifestyle Questionnaires (2010/2012: Time 1 and 2014/2016: Time 2) and the HRS data on APOEε4 alleles, linear regression models with a lagged dependent variable were performed to examine associations between productive and leisure activities with cognitive functioning at the follow-up time point, as well as the role of APOEε4 in these associations. Among all participants (n=7,605), employment, low or high-intensity volunteering, cognitive leisure activities, and social leisure activities were associated with higher levels of cognitive functioning. The presence of at least one ε4 allele was related to poorer cognitive functioning at Time 2. Among people without the APOEε4 allele (n=5,630), employment, high-intensity volunteering, cognitive/social leisure activities were significantly associated with cognitive functioning. Among people with at least one APOEε4 allele (n=1,975), low-intensity volunteering, and cognitive/physical leisure activities were significantly associated with better cognitive functioning. We found that employment, civic engagement, and leisure activities all contribute to cognitive health, although the benefits may be restricted to low-intensity volunteering and cognitive/physical leisure activities among individuals with at least one APOEε4 allele who are known to be inherently at a greater risk of cognitive problems, highlighting an avenue to a relatively easily-implementable strategy for cognitive health promotion.

